# Child Birth—Early Rising after

**Published:** 1915-11

**Authors:** E. C. Cartledge

**Affiliations:** Healy Building, Atlanta, Ga.


### CHILD BIRTH—EARLY RISING AFTER.

By E. C. Cartledge, M. D.

The chief reason for keeping a patient in bed two weeks approximately, after childbirth is: To avoid present and subsequent uterine prolapse; just as we all direct that the mother must not remain lying on her back all the time, but must rest upon both sides, including Sims' posture, in order that her uterus may not become set and fixed in any one position, retro or lateral, etc. We are sometimes forgetful in not instructing patients in this matter agreed upon by all.

Much more do I think we fail to remember the very great value of the knee-chest posture to be taken each retiring or reclining hour after having been on feet during day for the five or six weeks of puerperium. The value of this knee-chest posture was impressed upon me by two cases of mine. Both patients had disobeyed instructions. One was upon 10th day, dressed and trying to remain up all she could in order to regain her strength the faster, as she argued, in her hurry to leave the city.

Her uterus was very low upon examination. She was very nervous and acknowledged that she was becoming weaker instead of stronger since sitting up three or four days. But putting her back to bed and having her use the knee-chest posture and then sleep preferably and largely in Sims' position until the replacement was well established, she very soon felt well and the pelvis became normal.

The other case remained in bed her two weeks, but after that, neglected her instructions to arrive at normal living by slow degrees, gradually leaving her bed, and not remaining up entire day until four to six weeks should pass, according as her feeling and strength would invite her progress. But when her two weeks were passed and her nurse dismissed, she did not get a housekeeper as directed, but left her bed altogether and took up her usual task of doing all her housework. She sent for me three weeks after her confinement—one week after leaving her bed, and she was very weak and nervous and wondered why she wasn't gaining strength. I explained again to her how she must make haste slowly—undue haste making waste.

Her uterus was down—the cervix to the vulva. She came all right, with a proper involution and restoration of positions and relations in her pelvis, by putting her back in bed a week, using the knee-chest posture after each getting up on her feet in response to nature's calls and continuing its use at bed time for five or six weeks or until uterus and pelvic organs were normal. I try to impress the truth upon these patients by reminding them how the womb weighs about two pounds at baby time and how it must take time for it to reduce in size to two ounces from two pounds.

The expression "falling of the womb" impresses most women and drives them to better obedience. These two cases "gone bad" and then so easy of relief and recovery impress us with one of the reasons why so many women never quite have their health again after child-birth, and how easy it is to avoid this item of possible trouble. Since my experience with these two cases I have changed a bit my care of lying-in mothers. I examine them about two and four weeks after confinement to see position of uterus. Knowing the different degrees of vitality and vigor in women, I do not make them all alike remain in bed for any set time or stereotyped period, but let each one get out of bed partly, i. e. begin the graduated process of getting up, upon the fifth to twentieth day or just as they feel the desire and capacity for getting up, always guarding them with a certain amount of suspicion that they may presume upon the privilege I have allowed them and make haste to their defeat.

Surely it is right to individualize these recent mothers knowing the knee-chest posture save us from the only harm that can come from being on their feet early and much—except, of course, the harm there is always in that effort and exercise which approaches fatigue.

To avoid their being up too much from their bed, I explain to them that they must avoid any approach to fatigue, that they sit up only when they are tired of the bed and they get up to rest. We have all seen recent mothers who were unable to leave their bed for three to five weeks. We also see those who have always remained in bed nine days, a popular period of time with some. Others we see, of the stronger athletic woman, who have left their bed on fourth or fifth day and no harm come of it.

Therefore, I think it not just reasonable for the doctor to have any stated interval of time, any set period, during which the recent mother must remain in bed; but each mother as she may feel the strength, and the tire of her confinement, may be allowed to get up enough to rest herself, beginning anywhere from fifth to twentieth day and being properly instructed in the use of the genu-pectoral posture. We are reminded that it is becoming the approved habit among us to allow our operative cases up and out of bed much earlier than formerly. I allowed a recent appendectomy case to walk seven blocks two hours before his week was up.

Especially is this found better and really necessary in old patients who need every item in their favor, e. g. old prostatic cases.

What I have said is upon the presumption that two weeks is the period of time these patients are kept in bed usually and rather as a rule by most of us. I was so taught and have so seen it practiced here. It may be to some extent a provincialism with us.

I was taught to have these mothers assisted up to sitting position upon the commode from the very first.

I have seen no reason for changing this practice. I see many who use bed pans and do not allow this getting up for a few days.

The real thought that prompted this little paper is that I am no longer afraid to allow my recent mother to get up as early as they feel like doing so.

Healy Building, Atlanta, Ga.

